# Pyramiding wheat pre-harvest sprouting resistance genes in triticale breeding

**DOI:** 10.1007/s11032-022-01327-3

**Published:** 2022-09-22

**Authors:** Odile Moullet, Gemma Díaz Bermúdez, Dario Fossati, Cécile Brabant, Fabio Mascher, Arnold Schori

**Affiliations:** 1 Plant Breeding and Genetic Resources, Agroscope Changins, CH-1260 Nyon, Switzerland; 2 Present Address: Saint-Genis-Pouilly, France

**Keywords:** Triticale, Breeding, Pre-harvest sprouting, *TaPHS1*, *TaMKK3*, *TaQsd1*

## Abstract

Pre -harvest sprouting (PHS) is an important problem in cereal production reducing yield and grain quality. After decades of improvement, triticale remains particularly susceptible to PHS but no resistance genes or QTLs were identified so far in this species. As wheat shares the A and B genomes with triticale, wheat PHS resistance genes can be introgressed into triticale genome by recombination after interspecific crosses. In this project, three PHS resistance genes have been transferred from wheat to triticale by marker-assisted interspecific crosses, followed by four backcrosses. The gene *TaPHS1* from the 3AS chromosome of cultivar Zenkoujikomugi (Zen) and the *TaMKK3* and *TaQsd1*, respectively located on the 4AL and 5BL chromosomes derived both from cultivar Aus1408, were pyramided in the triticale cultivar Cosinus. Only the *TaPHS1* gene increases consistently the PHS resistance in triticale. The lack of efficacy of the other two genes, especially *TaQsd1*, could be the result of an imperfect linkage between the marker and the gene of interest. The introduction of PHS resistance genes did not alter agronomic nor disease resistance performances of triticale. This approach leads to two new, agronomically performant and PHS-resistant triticale cultivars. Today, two breeding triticale lines are ready to enter the official registration process.

## Introduction

Triticale (x*Triticosecale* Wittmack) is a self-pollinating cereal originated from artificial allopolyploidizations between the two related species, *Triticum* spp. and *Secale cereale* L. Most triticale cultivars are hexaploid with a genomic constitution of 2n = 6x = 42 chromosomes (AABBRR) combing the AABB genome of *Triticum* spp. with the RR genome of rye. The synthetic hybrid combines the high yield potential and the grain quality of wheat with the adaptability, vigor, and resistance to abiotic and biotic stresses of rye. Triticale is cultivated worldwide on almost four million hectares and nearly 90% of the production is concentrated in Europe (FAOSTAT 2020). The crop is primarily used on-farm as feed for pigs, poultry, and ruminants. Human consumption of triticale is marginal because the flour shows relatively poor aptitude for bread making (Naeem et al. 2002). Yet, triticale can be used for the preparation of cakes, cookies, and noodles (McGoverin et al. 2011), or in the brewing industry (Glatthar et al. 2002). Recently, triticale was intensively investigated as a potential energy crop (Petersen et al. 2020).

The first triticale cultivars released in the 1970s were characterized by elevated diseases resistances, but low fertility with high susceptibility to ergot, low grain yield, low specific weight, shriveled kernels inducing high protein content, excessive height combined with lodging, and the elevated sensibility to PHS (Oettler 2005). After 50 years of breeding, most early problems have been resolved except for the PHS sensitivity. Modern triticale cultivars are agronomically competitive with wheat and superior to wheat in terms of grain yield under biotic and abiotic stresses (Blum 2014). The high level of diseases resistance is regularly challenged by the emergence of new races in pathogen populations (Mergoum et al. 2019; Rodriguez-Algaba et al. 2020).

Today, PHS remains a persistent problem in triticale and in other cereal crops. It corresponds to the germination of grains in the ear prior to harvest (Finch-Savage and Leubner-Metzger 2006). PHS infers on seed germination and alters nutritional and quality properties (Lemmens et al. 2019). PHS is hold responsible for an estimated $ 1 billion annual loss worldwide (Black et al. 2006).

In cereals, PHS is controlled by both genetic factors and environmental conditions. During the late maturity stage, an elevated relative humidity, warm temperatures, and a reduced partial oxygen pressure stimulate grain germination (Finch-Savage and Leubner-Metzger 2006). With the changing global climate and the predicted increase of temperature and extreme weather events, the occurrence of PHS may even increase (Singh et al. 2021). Seed dormancy, the capacity of the seed to avoid germination under normally conducive environmental conditions for seed germination (Baskin and Baskin 2004), is recognized as one of the main factors in resistance to PHS (Fidler et al. 2018). The hormonal balance between the antagonists gibberellic acid (GA) and abscisic acid (ABA) complex pathways regulates seed dormancy (Gao and Ayele 2014; Tai et al. 2021).

Several studies have identified genes and QTLs in common wheat conferring relative tolerance PHS (Ali et al. 2019; Singh et al. 2021). However, only few QTLs or genes have been effectively validated in different germplasm groups and in multiple environmental conditions (Vetch et al. 2019).

The *TaPHS1* (or *TaMFT-3A*) is a major gene involved in PHS resistance (Mares and Mrva 2014). It has been located on wheat chromosome 3AS and was identified in the highly dormant wheat variety Zenkoujikomugi (Zen) (Mori et al. 2005). This gene encodes a homolog of a phosphatidylethanolamine-binding protein responding in *Arabidopsis* to ABA and GA signals (Xi et al. 2010). Functional sequence variations in *TaPHS1* have been characterized in diploid, tetraploid, and hexaploid wheats (Liu et al. 2021). Diagnostic markers for the gene are available (Jiang et al. 2018; Wang et al. 2020). A second important gene influencing PHS is *TaMKK3* located on the chromosome 4AL (Tan et al. 2006; Shorinola et al. 2016; Torada et al. 2016). This gene encodes a mitogen-activated protein kinase (MAPK) kinase 3 involved in Arabidopsis in the phosphorylation of important proteins of the ABA signal transduction pathways (Danquah et al. 2015).

The wheat homeologs of *Qsd1* in barley (quantitative trait locus on seed dormancy 1), *TaQsd1*, encode an alanine aminotransferase expressed specifically in embryos (Onishi et al. 2017). The gene, located on the chromosomes group 5, regulates the level of seed dormancy (Abe et al. 2019).

The aim of this study was to assess the efficiency of PHS resistance genes transferred from wheat to triticale on pre-harvest sprouting and seed dormancy in triticale. For this, we first compared different methods for the evaluation of PHS resistance in cereals to identify the easiest and fastest but still reliable method in a triticale breeding context. In a second step, we have introduced three wheat resistance genes from the A and B genomes (*TaPHS1*, *TaMKK3*, and *TaQsd1)* into triticale by marker-assisted interspecific backcrossing. In a third step, we evaluated the impact of wheat PHS resistance QTLs and combinations of QTLs on both the level of PHS and on seed germination in triticale. Our final aim was to create new triticale varieties with an increase level of PHS resistance while maintaining an elevated yield and disease resistance potential.

## Materials and methods

### Plant material and origin of PHS resistance QTLs

The Japanese red spring wheat variety Zenkoujikomugi (Zen) bears the PHS resistance QTL *TaPHS1*, located on chromosome 3AS (Mori et al. 2005). The Australian wheat variety AUS1408 carries also the *TaPHS1* (Lin et al. 2018) and two other resistance QTLs *TaMKK3* on chromosome 4AL and *TaQsd1* on chromosome 5BL (Tan et al. 2006). Cosinus is a triticale variety bred by KWS LOCHOW GMBH (Germany) and registered in 2010.

### Markers of PHS resistance QTLs

For all three QTLs, we dispose of tightly linked molecular markers. The *TaPHS1* of cultivar Zen can be detected by the SSR marker *barc57* (primers 5’-GCG ACC ACC TCA GCC AAC TTA TTA TGT-3’ and 5’-GCG ACC ACC TCA GCC AAC TTA TTA TGT-3’) (Kottearachchi et al. 2006). The sizes of the expected fragments are not mentioned in the original publication.

The PCR marker *ZXQ118* (primers 5’-CTG ACT GAT ATA CGG CAA TC-3’ and 5’-ATG TGA TTG GTT GAT CAA GCG-3’) validated by Zhang et al. (2008) identifies the QTL located on chromosome 4AL of Aus1408. In a preliminary test, we have validated the lengths of the DNA fragments in the resistant (Aus1408) and in the susceptible triticale parental line Cosinus.

The SSR markers *wmc118*, *barc59*, and *gwm497*, linked to the QTL of chromosome 5BL of Aus1408, as proposed by Tan et al. (2006) resulted non-polymorphic fragments compared to Cosinus. We opted therefore for the marker *wmc783* (primers 5’-AGG TTG GAG ATG CAG GTG GG-3’ and 5’-TCT TCC TTC TCC TGC CGC TA-3’) that maps between *barc59* and *gwm497* (Somers et al. 2004). This marker enabled us to distinguish the amplified fragments on 2.5% agarose gel from resistant Aus1408 and susceptible Cosinus. For all the three markers, the annealing temperature was 60 °C.

**Fig. 1:**
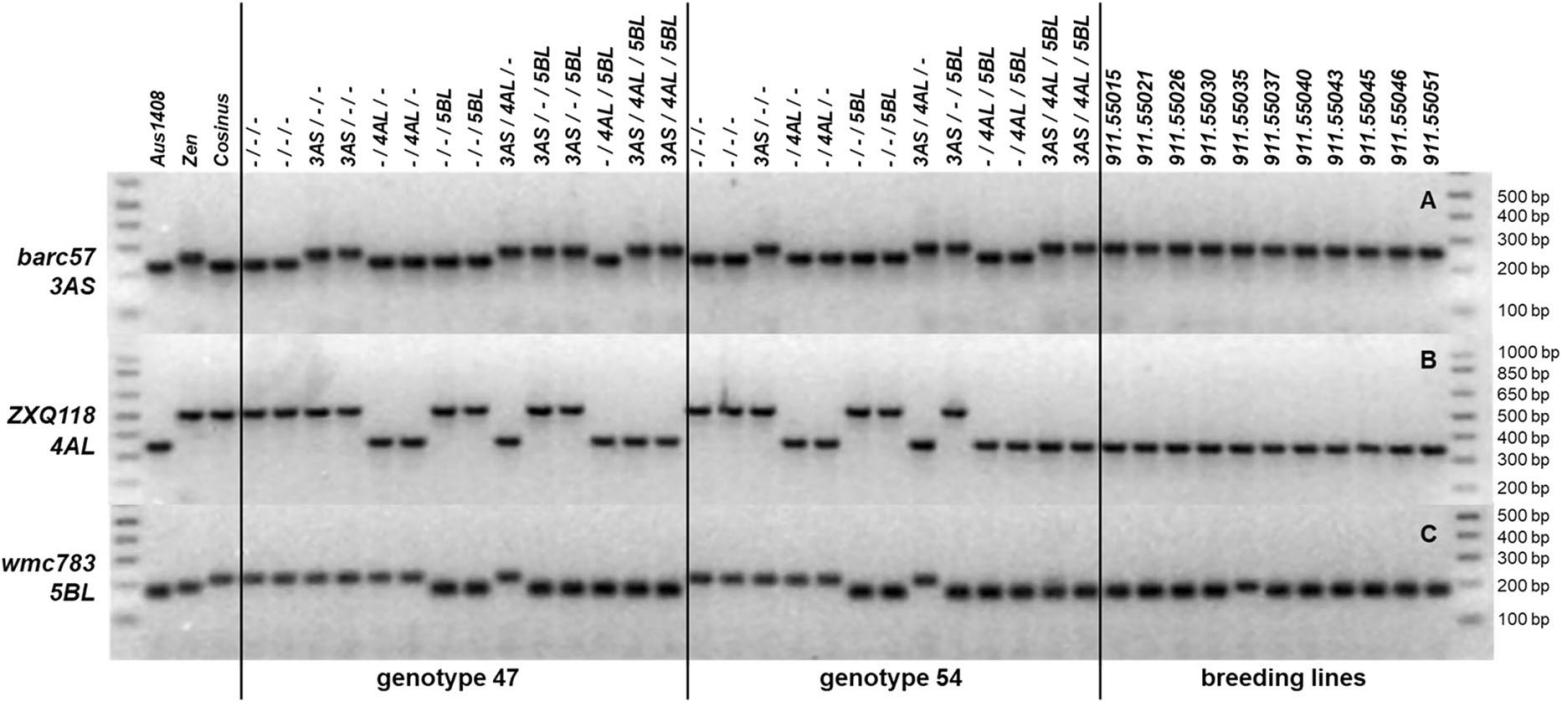
PCR profile showing the polymorphism between the parental lines (Aus1408, Zen, Cosinus), the lines with particular PHS resistance QTLs derived from the genotypes 47 and 54 and the breeding lines using the primers: A/ *barc57* for *TaPHS1* (3AS), B/ *ZXQ118* for *TaMKK3* (4AL) and *wmc783* for *TaQsd1* (5BL)

DNA extractions, PCR amplifications, and nucleic fragments analyses are described in Moullet and Schori (2014). The exact DNA fragments length of the PCR products were not determined. Usually, we only scored the polymorphisms on agarose gels (Fig. 1).

### Embryo rescue

Embryo rescue methods are used in interspecific crosses to raise plants from non-viable seeds. This approach also allows reducing the generation time by up to 2 months.

In the present, the developing caryopses were harvested from main spikes at 17 or 18 days after fecundation, surfaced-sterilized first in 100% ethanol for 30 s, and then in a solution of sodium hypochlorite containing 4% (w/v) chlorine and 0.4% tween 20 for 15 min. Subsequently, the seeds were rinsed five times in sterile distilled water. Immature embryos (2 to 3 mm size) were aseptically excised from young caryopses, placed scutellum-side up on plant induction MS medium (Murashige and Skoog 1962) supplemented with l-asparagine 150 mg/lt, thiamine hydrochloride 16 mg/lt, and AIA 0.1 mg/lt. After incubation for 5 days at 10 °C, the embryos were incubated at 23 °C for 3 to 7 days until the plantlets were grown to 1 to 2 cm long. These plantlets were immediately transplanted into soil.

### Transfer of PHS resistance genes into triticale

**Fig. 2:**
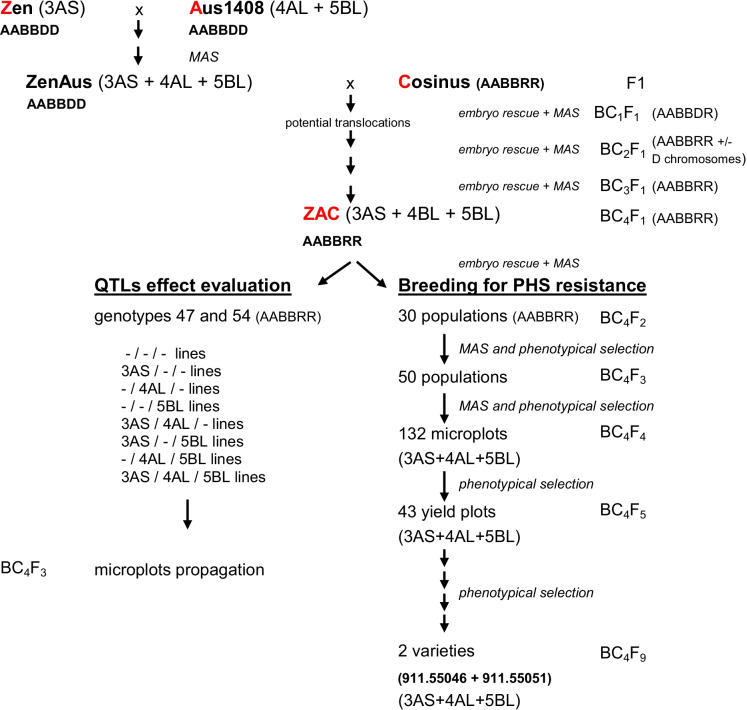
Diagram showing steps involved in the production of lines used for the evaluation of PHS resistance QTLs effect and for marker-assisted breeding for PHS resistance. 3AS, 4AL and 5BL represent the QTLs for PHS resistance located on the 3AS, 4AL et 5BL chromosomes. Except for the plants ZAC (3 heterozygous QTLs), the lines are homozygous for the mentioned QTLs

The transfer of PHS resistance genes from wheat varieties Zen and AUS1408 to triticale variety Cosinus is displayed in Fig. 2. In a first step, we have crossed Aus1408 and Zen in order to combine all three QTLs in the same wheat background (ZenAus). From 960 self-pollinated ZenAus plants F2, 8 homozygous plants for all three QTLs of interest were selected by marker-assisted selection (MAS). One hundred spikes out of these plants were fecundated with pollen of the triticale cultivar Cosinus to produce more than 1850 seeds. The germination faculty of 1000 hybrid seeds was assessed yet all tested seeds were non-viable. Eight plants (F1) have been recovered by embryo rescue on mature seeds. The hybrids were sterile but a subsequent fecundation of 50 emasculated spikes with Cosinus pollen produced 50 seeds. Using MAS, we could identify six plants (BC2F1) fully fertile carrying all three QTLs of interest. After a series of four backcrosses with Cosinus, we obtained the ZAC lines (**Z**en × **A**us × **C**osinus). In all backcrosses, plants were recovered using the embryo rescue technique. Rescued plants were used as female plants in the following backcross cycle.

Male and female plants for the crossing experiment were grown under controlled conditions in a phytotron after vernalization for 6 weeks in a cold chamber at 4 °C. By this procedure, we obtained two generations per year.

The progeny of two BC4F1 plants (namely line 47 and line 54) were analyzed with markers to identify those plants containing none (-/-/-), one (3AS/-/-, -/4AL/-, and -/-/5BL), two (3AS/4AL/-, 3AS/-/5BL, and -/4AL/5BL), or all three (3AS/4AL/5BL) homozygote QTLs. For most combinations of QTLs, two lines were obtained. Only one line could be obtained for the genotypes 3AS/-/- and 3AS/-/5BL of line 54, 3AS/4AL/- of both lines 47 and 54, and -/4AL/5BL of line 47. Seeds from single plants were multiplied in field microplots (BC4F3 in 2015).

### PHS resistance evaluation

**Fig. 3:**
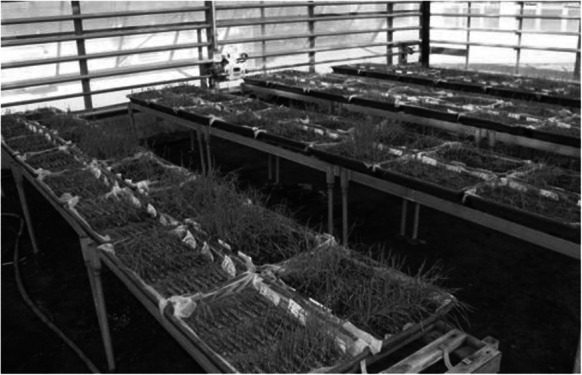
PHS assessment experiment. Almost 200 lines are tested in parallel in a shaded glasshouse. The picture shows emerging plantlets 14 days after the harvest

To evaluate PHS resistance (Kumar et al. 2010), ten intact spikes from ten plants of each genotypes from line 47 have been grown in the field and collected at full maturity. Quickly after harvest, the spikes were soaked for 5 h in tap water, then placed on a layer of 3.5 cm moist sand, and covered by jute bags in a shaded greenhouse at 20 to 30 °C (Fig. 3). An automatic watering system wetted the ears 7 times a day with 5 mm tap water.

**Fig. 4:**
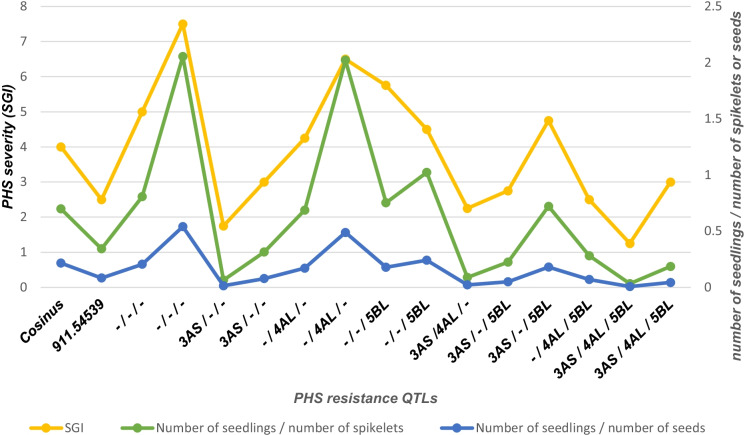
Comparison of methods for PHS resistance evaluation. The PHS resistance is expressed with the SGI data (yellow), the number of seedlings per number of spikelets (green) or per number of seeds (blue) after 8 days. The evaluated lines represent the progeny of the genotype 47

Three scoring methods for PHS resistance have been compared (Fig. 4) to find the most reliable and in the same time the most accurate method. The first method estimates the number of emerging plantlets on a visual scale from 1 to 9, with 1 for none and 9 for complete germination. The integration of the germination score with time gives the spike germination index (SGI) using the following equation:

$$SGI=\sum ({y}_{i}+{y}_{i+1})*({t}_{i}-{t}_{i+1})/2$$

where *y*_*i*_ is the score for PHS at the *i*th day and *t*_*i*_* − t *_*i*+*1*_ the number of days between two consecutive observations.

The second and the third methods were to count the proportion of germinated seeds per number of spikelets (method 2) or per number of seeds (method 3) after 8 days of incubation.

After evaluation, we opted for the visual estimation of germination with the calculation of the SGI every day over 14 days as the most accurate and quickest scoring method of triticale germination.

### Seed dormancy scoring

Dormancy was evaluated on freshly harvested seeds collected in the centre of the spike of each genotype. Overall, 50 seeds, namely two from 25 spikes were collected. The seeds were placed with the crease facing down on a thick filter paper in a Petri dish and 3 ml of sterile distilled water was added. Subsequently, the plate was incubated in the dark at either 10 or 30 °C. Every third day, 0.5 ml at 10 °C and 1 ml at 30 °C of sterile distilled water was added to keep the filter moist. Once a day at approximately the same time for 2 weeks, grains presenting 1 to 2 cm long coleoptile were counted and discarded. The dormancy of seeds were finally expressed as the weighted germination index (WGI). The WGI was calculated according to Nyachiro et al. (2002) using the following equation:

$$WGI=\{14*{n}_{1}+13*{n}_{2}+\dots +1*{n}_{14}\}/N$$

where *n* is the number of germinated seeds at day *n*_i_ while *N* is the number of total seeds and the integer is the daily weighting factor.

### Seed multiplication and agronomic evaluation

For the selection of PHS-resistant triticale cultivars, 300 seeds from 30 BC4F2 (2014) lines were sown in single rows in the field (Fig. 2). The presence of the three QTLs has been analyzed in 820 plants out of whom 50 single plants (BC4F3) were selected for the effective presence of resistance QTLs but also on phenotypical criteria such as diseases resistance, plant height, lodging resistance, tillering capacity, spike length, and fertility. In 2015, populations of 120 to 360 seeds per chosen line were grown in separate field plots. At this stage, 132 homozygous for the three QTLs (BC4F4) and phenotypically promising plants were chosen for the next generation. In 2016, one microplot per selected plant was grown. The visual selection completed with an approximate yield, a disease resistance evaluation (glume blotch and stripe rust) and pre-harvest sprouting resistance scoring allowed the selection of 43 lines (BC4F5). The following year, these lines were evaluated in performance trial in the field (two replicates of each line in randomized yield plots of 4.7 m × 1.5 m, 400 seeds m-2). The final process of selection from 2018 to 2020, involved six Swiss locations field trials containing three plots (4.7 m × 1.5 m, 400 seeds m-2) per line grown in a randomized complete block design. In parallel, a disease assessment nursery under artificial inoculation allowed an accurate disease resistance evaluation. Each disease was evaluated separately with artificial inoculations for powdery mildew, stripe rust, leaf rust, Stagonospora glume blotch, and fusarium head blight resistance (Michel 2001). The most promising lines, combining PHS resistance, disease resistance and yield performance were compared with the recently released triticale varieties Balino, Trialdo, Larossa, and Cosinus.

### Statistical analysis

Data were systematized in spreadsheets (Excel 2016). Statistical analyses of data were performed with R (R Core Team 2021).

#### Comparison of PHS evaluation methods

The relation between the three PHS evaluation methods, namely either estimating or counting the number of germinating grains, was studied with a Pearson correlation analysis using the package “stats” included in R. The necessary assumptions on normality, linearity and absence of outliers ascertained beforehand using the Shapiro-Wilks normality test after transformation (squaring) of the data.

#### Influence of resistance genes on PHS and dormancy

The influence of the different PHS resistance genes and their combination on sprouting was analyzed with linear mixed models (LMM) using the “lme4” and the “lmerTest” packages (Bates et al. 2015; Kuznetsova et al. 2017). We used this model to estimate the effects at different levels (QTLs and genotypes levels) with respect to the mean SGI values taking as a reference the results obtained with Cosinus. The model includes years and genotypes as random effects. Finally, the model used was adapted from Piepho et al. (2004):

$$\begin{array}{l}{SGI}_{vg}=\mu +{a}_{v}+ {b}_{g}+{R}_{vg}+{\epsilon }_{vg}\\ v=breeding\_line1\dots .breeding\_10 g=Genotype\ 47,Genotype\ 54\end{array}$$

where ${SGI}_{vg}$ is the observed value; $\mu$ is the overall mean; ${a}_{v}$ is the fixed effect of the QTL or the combination of QTLs; ${b}_{g}$ is the fixed effect of each genotype (line 47 or line 54); ${R}_{vg}$ is the random effect between the genotype and the years; and ${\epsilon }_{vg}$ is the residual (random and fixed) error of $SGIvg$. Since differences between QTLs and combinations of QTLs are significant, the pairwise comparisons of the means for the different QTLs were calculated as estimated marginal means (EMMs) using the “Emmeans” package (Russell 2021). Subsequently, the comparisons between the EMMs were done with Tukey’s post hoc test using the “pwpp” function.

## Results

### Comparison of methods of evaluation of pre-harvest sprouting

For the comparison of three methods for evaluation of PHS, only progenies of genotype 47 containing none, one, two, or all three homozygous QTLs were tested. Cosinus and the breeding line CH-911.54539 were used as standards. The methods tested included (1) the estimation of emerging plantlets by visual scoring every second day during 8 days, expressed as SGI, (2) the counting of the proportion of germinating seeds per number of spikelets after 8 days, and (3) the counting of the proportion of germinating seeds per number of seeds. Figure 4 shows the results obtained for each genotypes with all three scoring methods. The correlation between the SGI with the other two scoring methods is shown in Table 1. All three methods were very similar, showing a highly significant correlation coefficient (*r* = 0.95 and *r* = 0.94) between SGI and the other two scoring approaches. In the following, we have opted for the easier and quicker SGI method.

**Table 1:** Comparison of methods for PHS resistance evaluation. Table of correlation analysis for the variables number of seedlings per number of spikelets or number of seeds versus the observed SGI

Methods	T	DF	Confidence interval (95%)	*p* value	*r*	*R*^2^
Spikelets	11.94	14.00	[0.87, 0.98]	< .001	0.95	0.90
Seeds	11.94	14.00	[0.87, 0.97]	< .001	0.94	0.88

### Effect of resistance QTLs on pre-harvest spouting

**Fig. 5:**
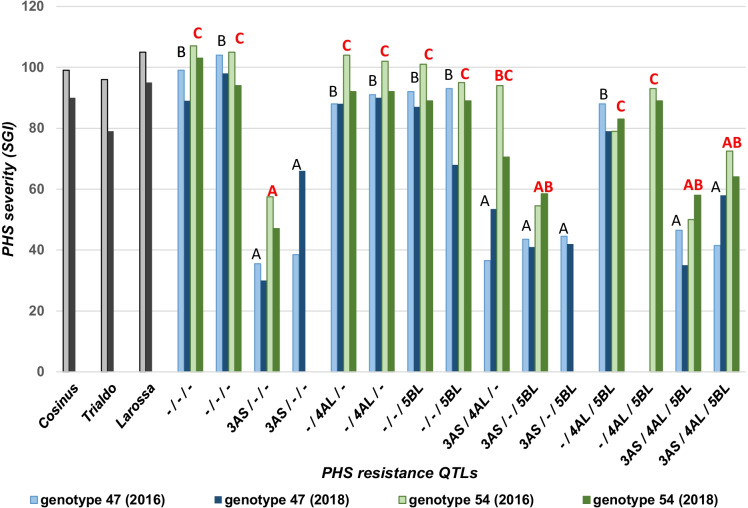
Effect of wheat PHS resistance QTLs on Triticale sprouting. SGI is calculated on data scored during 14 days. PHS resistance on genotype 47 (blue) and 54 (green) were evaluate in 2016 (light bars) and in 2018 (dark bars). Comparisons of means by genotype with letters (genotype 47 in black and 54 in red). Means that do not share a letter are significantly different. Grouping information using Tukey’s method and 95% confidence

Pre-harvest sprouting was investigated on the progeny lines of both genotypes, line 47 and line 54, and on triticale varieties Cosinus, Trialdo, and Larossa for comparison. The analysis was done on 10 intact mature spikes. In order to obtain more accurate SGI in this experiment compared to the previous one, we visually assessed PHS every day for 14 days resulting in higher SGI data. Susceptible genotypes germinate completely within 5 to 7 days while in resistant ones only few seedlings were visible after 21 days. The results are presented in Fig. 5. The outcome of the LMM analysis shows that the effects of the QTLs are statistically significant, while the effect of parental genotype (notably line 47 and line 54) were not significant (Table 2). Table 3 shows the pairwise comparisons of the effect of the QTLs and combinations of QTLs on PHS. It became evident that all lines containing the QTL on chromosome 3AS show reduced PHS. The two other QTLs have also an effect, but weaker and less consistent. Lack of 5BL QTL efficiency may result from imperfect linkage between the *wmc783* marker and the QTL of interest.

**Table 2:** Anova study showing the effects of the QTLs and genotypes. In the table, we have the source of variation (source), the sum of squares (Sum sq), the mean of squares (Mean sq), the degrees of freedom (DF), and the value of the *F*-statistic (*F* value) with its associated *p*-value (Pr(> *F*))

Source	Sum Sq	Mean Sq	NumDF	F value	Pr(> F)
Lines (QTLs)	26,557.80	2655.78	10.00	34.41	< 0.01***
Parental genotypes	386.30	386.31	1.00	5.01	0.15

Signif. codes: 0 ‘***’ 0.00 1 ‘**’ 0.01 ‘*’ 0. 05 ‘.’ 0.1 ‘’ 1

**Table 3:** Effect of resistance QTLs on pre-harvest sprouting. This matrix shows the estimated marginal means (EMMs) along the diagonal, *p* values in the upper triangle, and the differences of Emmeans in the lower triangle. Significant values *p < 0.05

	Cosinus	-/-/-	-/-/5BL	-/4AL/-	-/4AL/5BL	3AS/-/-	3AS/-/5BL	3AS/4AL/-	3AS/4AL/5BL	Larossa	Trialdo
Cosinus	[94.3]	0.992	0.995	1.000	0.698	< .0001*	< .0001*	0.0001*	< .0001*	0.996	0.978
-/-/-	− 5.324	[99.6]	0.392	0.846	0.056	< .0001*	< .0001*	< .0001*	< .0001*	1.000	0.351
-/-/5BL	5.104	10.429	[89.2]	0.999	0.978	< .0001*	< .0001*	< .0001*	< .0001*	0.545	1.000
-/4AL/-	1.810	7.135	− 3.294	[92.5]	0.744	< .0001*	< .0001*	< .0001*	< .0001*	0.933	0.995
-/4AL/5BL	10.486	15.810	5.382	8.675	[83.8]	< .0001*	< .0001*	0.0021*	< .0001*	0.107	1.000
3AS/-/-	47.031	52.356	41.927	45.221	36.546	[47.3]	1.000	0.933	0.947	< .0001*	< .0001*
3AS/-/5BL	45.411	50.736	40.307	43.601	34.926	− 1.620	[ 48.9]	0.984	0.994	< .0001 *	< .0001 *
3AS/4AL/-	38.667	43.991	33.563	36.857	28.181	− 8.365	− 6.744	[55.6]	1.000	< .0001*	0.0118*
3AS/4AL/5BL	40.917	46.242	35.813	39.107	30.432	− 6.114	− 4.494	2.250	[53.4]	< .0001*	0.0003*
Larossa	− 5.661	− 0.337	− 10.765	− 7.472	− 16.147	− 52.693	− 51.072	− 44.328	− 46.578	[100.0]	0.628
Trialdo	7.165	12.490	2.061	5.355	− 3.320	− 39.866	− 38.246	− 31.501	− 33.752	12.827	[87.1]

### Effect of PHS resistance QTLs on seed dormancy (WGI)

The seed dormancy was investigated on the same genotypes. Seed dormancy is expressed as Weighted Germination Index (WGI) (Table 4). The WGI was scored at two temperatures, where 10 °C being the dormancy-breaking temperature for most cereals and 30 °C allowing only non-dormant seeds to sprout. The WGI was calculated on data collected during 17 days. In 2016, the WGI at 30 °C of two lines was not determined because of fungal contaminations of the seeds. For the cultivar Trialdo, 10 °C breaks only partially the dormancy, whereas for all other lines at this temperature, almost full germination occurs within 2 weeks. For the most dormant genotypes, only one seed out of 25 seeds was growing at 30 °C during experimental time.

**Table 4:**
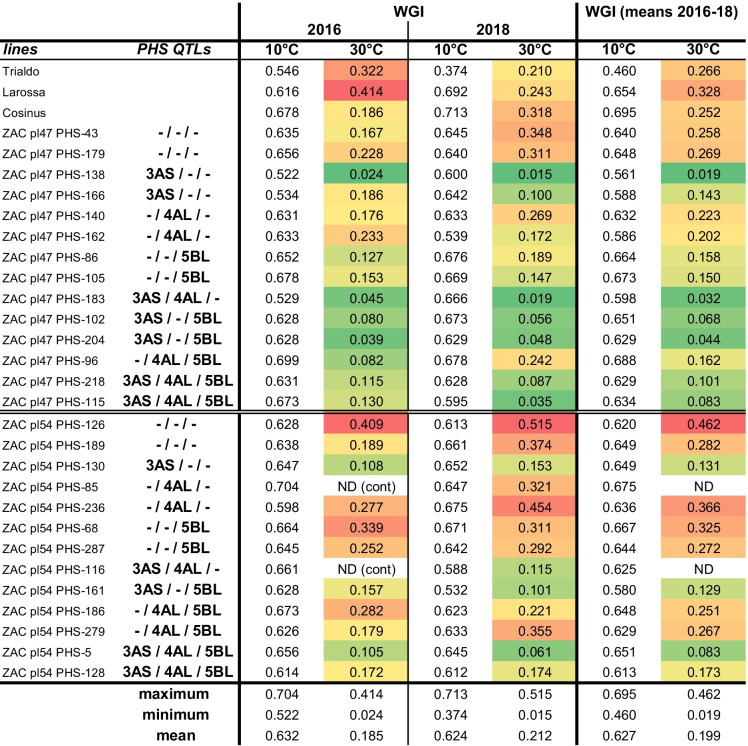
Effect of wheat PHS resistance QTLs on Triticale dormancy. WGI is calculated at 10 and 30 °C from lines grown in the field in 2016 and 2018. *ND*, not determinate; *cont*, contaminated

The shade of the color represents higher (red) or lower (green) WGI values

**Fig. 6:**
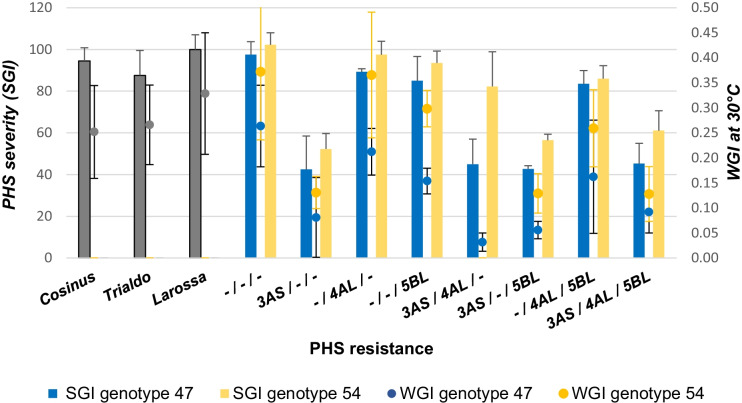
Effect of wheat PHS resistance QTLs on Triticale sprouting. SGI (bars) and WGI at 30 °C (dots) represent the average data from each QTLs combinations collected in 2016 and 2018 with genotype 47 (blue) and 54 (yellow). Vertical bars: standard deviation

The means values for the SGI and WGI of every combinations of QTLs for the 2 years of analysis was calculated to easier evaluate the effects of each QTLs (Fig. 6).

**Fig. 7:**
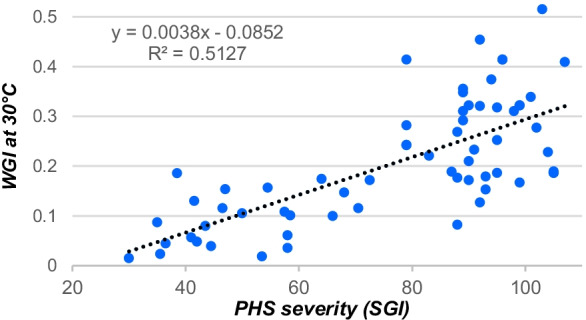
Scatterplot showing the Pearson’s correlation between PHS severity (SGI) and WGI at 30 °C

Pearson’s correlation analysis was carried out between SGI and WGI showing a significant and positive degree of association (*r* = 0.72, *t* (62) = 8.08, *p* > 0.001), i.e., the higher the WGI value at 30 °C, the higher the SGI value and vice versa (Fig. 7).

### Breeding for PHS resistance

**Fig. 8:**
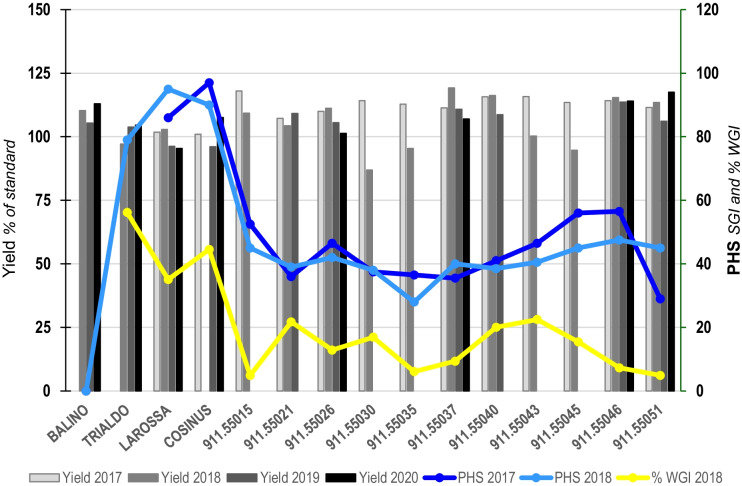
Breeding for PHS resistance. The bars represent yield expressed in percentage of standard varieties (Balino, Trialdo, Larossa, and Cosinus) during 4 years of experiments (2017 to 2020). The blue curves show the PHS resistance expressed in SGI and the yellow curve is the percentage of WGI when WGI at 30 °C is compared to WGI at 10 °C (100%)

The final aim of our project is to create new commercial cultivars of triticale resistant to PHS and agronomically performant. The most promising triticale lines containing the 3AS, the 4AL, and the 5BL QTLS for resistance to PHS were extensively evaluated during a final process of breeding (2018 to 2020) involving six locations field trials (Fig. 8 and Table 5).

**Table 5:** Agronomical performance of new PHS-resistant Triticale cultivars evaluated in 2020. Yield after sorting is expressed as % of the mean of controls cultivars (Balino, Trialdo, Larossa and Cosinus). *TKW*, thousand-kernel weight; *HLW*, hectoliter weight

			Balino	Trialdo	Larossa	Cosinus	911.55026	911.55037	911.55046	911.55051
Yield	Absolute	dt/ha	98.1	90.7	83.3	94.2	88.2	93.4	99.4	101.6
	After sorting	%	126.6	102.5	97.7	110.1	107.8	114.3	123.9	126.4
	Protein	dt/ha	11.07	10.08	9.81	10.08	8.86	9.74	10.72	11.47
Maturity		days	136.5	134.8	134.3	136.0	137.5	131.7	133.3	133.7
Height		cm	113.9	105.6	111.9	120.8	119.4	110.0	123.9	121.4
Lodging		*	1.0	1.0	1.0	1.1	1.0	1.1	2.4	1.1
TKW		g	53.1	47.7	46.5	46.5	42.5	42.8	45.9	49.4
HLW		kg/hl	74.4	74.2	70.9	73.2	73.1	75.9	76.5	75.6
Powdery mildew	Natural	*	1.0	1.0	1.0	4.0	1.0	3.0	3.0	2.7
	Artificial	*	4.0	4.0	5.3	4.0	5.7	5.3	5.0	4.7
Stripe rust	Natural	*	1.0	1.0	1.0	2.3	1.0	1.0	1.0	1.0
	Artificial	*	1.0	1.3	1.0	4.3	3.0	1.0	1.0	1.0
Leaf rust	Natural	*	3.3	2.6	3.9	2.0	1.6	1.7	2.3	2.4
	Artificial	*	1.7	1.0	2.3	1.7	1.3	1.0	1.7	2.3
Septoria nodorum	Natural	*	2.0	2.3	3.0	3.7	3.7	4.0	3.0	2.3
	Artificial	AUDPC	77	58	58	141	131	73	96	73
Fusarium	Artificial	*	1.0	2.5	2.3	2.0	2.0	2.3	2.0	2.3

^*^Note: scale is 1 to 9 with 1 denoting no symptoms

The SGI was calculated in 2016 and in 2018. The % WGI curve represent the percentage of WGI at 30 °C compared to the WGI at 10 °C and was assessed in 2018 only (Fig. 8).

The selected lines (911.550xx) containing the three QTLs for resistance to PHS were compared to recent performing varieties named Balino, Trialdo, Larossa, and Cosinus respectively registered in 2019, 2012, 2014, and 2010. Several agronomical breeding traits (Table 5) were evaluated under natural growing environment.

The fungal diseases resistances were also estimated under artificial inoculation conditions in a field nursery.

The resulting lines (911.55026, 911.55937, 911.55046, and 911.55051) are resistant to PHS (SGI) and display high dormancy (WGI). All derived seeds germinate promptly after 2 months of storage at room temperature. The yield, the resistance to lodging, the thousand-kernel weight, the hectoliter weight, and the disease resistance are similar to the best recent Swiss cultivars. The line 911.55051 is slightly better than Balino in terms of absolute or protein yield and reaches heading stage approximatively 3 days before the control cultivar. That line is longer than Balino but shows almost no lodging. Concerning the diseases resistance, 911.55051 is slightly more susceptible to powdery mildew and fusarium head blight.

## Discussion

### Obtaining plant materials

PHS is a major problem in most cereals species. Several QTLs for PHS resistance were identified in wheat (Ali et al. 2019). Hexaploid triticale shares the genomes A and B with bread wheat. The transfer of genetic information from wheat to triticale is feasible by interspecific crosses (Saulescu et al. 2011).

The crossability and the emergence of F1 hybrids are highly depends on triticale cultivars involved in the crosses (Nkongolo et al. 1991). In this study, F1 hybrid seeds were easily produced by crossing the triticale genotype Cosinus as pollen donor line and the emasculate F2 hybrid wheat lines (Zen × Aus1408) containing the three PHS resistance QTLs. The shriveled seeds produced were sterile and embryo rescue on mature caryopses was required to generate F1 hybrid plants. A reciprocal cross, possibly increasing the germination rate (Hills et al. 2007), was impossible to achieve due to the low availability of homozygous wheat plants. The auto-incompatibility of F1 hybrid plants constrained to transfer manually triticale pollen to generate BC1F1 seeds. At this stage, the hybridization rate (< 2%) was much lower than for the initial cross (> 80%) and embryos rescue was performed on immature seeds to create plants for the next generation. Embryo rescue is a powerful technique to recover potential seedlings from kernels but also to speed up the process of backcrossing by shorten the grain-filling period. The production of the next generation female plants through embryos rescue should not select non-dormant genotypes. Isolated wheat embryos expressed reduced dormancy during grain-filling (Gerjets et al. 2010). As almost all embryos regenerate fully normal plants, the incubation at 10 °C of the embryos overcomes dormancy requirement, which is crucial to reach our final objectives. The time saving provided by embryo rescue (1 up to 2 months per generation) is important in the context of backcross. A disadvantage of this breeding method is the time necessary to complete the whole process (five or six cross generations to reach a level of homozygosity close to the elite parental lines and one generation to obtain homozygous plants for the favorable QTLs) at the end of which the recurrent parent could be surpassed by recent releases.

After four backcrosses, triticale lines should theoretically conserve less than 3% of the wheat genome but remain phenotypically distinguishable. Cytogenetic analysis of the progeny issue from interspecific hybridization between wheat and triticale reveals high levels of translocations in the hybrids plants (Lukaszewski and Gustafson 1983; Jeberson et al. 2021).

### Comparison of methods for pre-harvest sprouting evaluation

Breeders have developed a series of methods for the detection of PHS resistance in cereal including artificial sprouting of intact spike, germination tests, natural, or artificial weathering field trials or Hagberg Falling Number (Derera 1989; DePauw et al. 2012).

Germination increases the α-amylase activity in cereal seed. The Hagberg Falling Number (FN) assay reveals the viscosity of a flour suspension in water directly link to the enzymatic starch degradation triggered by sprouting. The FN method is internationally accepted for the measurement of grain quality reduction related to the sprouting damage. With our tested lines derived from the genotype 47 or 54, FN is not correlated with PHS severity estimated with SGI or WGI (data not shown). In triticale, low FN is not indicative of high α-amylase activity (Dennett et al. 2013). The FN is also not well correlated with dormancy (Alaru et al. 2008) or mature spikes sprouting (Sodkiewicz 2002). In some wheat cultivars, FN is also affected by various factors other than sprouting damage (Barnard and Smith 2012; Mares and Mrva 2008). In a genome-wide association mapping (GWAS) study on wheat germplasm, sprouting scores and FN were not strongly correlated and no overlapping quantitative trait nucleotides (QTN) based on FN and PHS were detected (Martinez et al. 2018).

While looking for an accurate, easy, and fast method, we have compared different counting and estimation methods on germinating ears. A simple 1–9 estimation of the extent of germination of the ears was the best approach, meeting all our requirements and allows the calculation of the SGI. It takes into account the extent of ear germination over the whole observation period.

### Effect of PHS wheat QTLs on triticale

In recent year, several individual genes and QTLs affecting seed dormancy were characterized and localized on all wheat chromosomes (Ali et al. 2019; Tai et al. 2021). Only a few genes are consistent in multiple studies across many germplasm and environments (Vetch et al. 2019) including the *TaPHS1* (Nakamura et al. 2011), the *TaMKK3* (Torada et al. 2016), the *Tamyb10*, closely associated with the red grain color genes (Himi et al. 2002) and the *TaVp1* (Nakamura and Toyama 2001; McKibbin et al. 2002). Two of these genes, the *TaPHS1* and the *TaMKK3*, referred here as 3AS and 4AL, were introduced into triticale genome by marker-assisted backcrosses. Wheat map comparison (Somers et al. 2004, GrainGenes (http://wheat.pw.usda.gov/GG3)) shows that the third QTLs from the 5BL chromosome of the germplasm Aus1408 (Tan et al. 2006) is located in the region of *TaQsd1* genes (Onishi et al. 2017; Wei et al. 2019). The wheat orthologous loci of *Qsd1* gene, initially found in barley, significantly increase the seed dormancy period (Abe et al. 2019).

The map-based cloning of *TaPHS1* from the chromosome 3AS and the sequencing of BAC contig of the entire QTL region shows that the SSR marker *barc57* is closely link to the gene (Liu et al. 2013). The robust, allele-specific marker *ZXQ118* mapping in the middle of the 4AL QTL peak, is strongly associated with dormancy (Zhang et al. 2008). The third marker used, the *wmc783* was found on wheat maps (Somers et al. 2004). The *TaQSd1* on the chromosome 5BL accounts for a minor proportion of PHS variations (Vetch et al. 2019) and closely link markers have been found only recently (Onishi et al. 2017; Wei et al. 2019).

In Zen germplasm, three QTLs associated with grain dormancy have been discovered on chromosomes 3A, 4A, and 4B (Mori et al. 2005), the *QPhs.ocs-3A.1* being the more reliable one. Kottearachchi et al. (2006) confirm the importance of the 3AS QTL from Zen but with the 4AL QTL, no difference in germination data was highlighted. For our study, the 4AL QTL from Aus1408 was favored.

In our experiments, not enough data have been collected to evaluate in details the precise epistatic interaction between the QTLs introduced into triticale. The effect of these QTLs depends on genotypes. The genotype 54 is generally more susceptible to PHS than the genotype 47. Alone or in combination with other QTLs, the *QPhs.ocs-3A.1* (*TaPHS1*) from the hard red wheat cultivar Zen (Mori et al. 2005) strongly increases the level of PHS resistance of both triticale genotypes 47 and 54 (Fig. 6). The two other QTLs (*TaMKK3* and probably *TaQsd1*) originated from white-grained cultivar Aus1408 (Tan et al. 2006) reduce slightly the PHS and no additive effects could be establish with the SGI.

The quantification of inherent dormancy through WGI analysis shows the same significant effect of the *TaPHS1* gene. The *TaQsd1* gene from the chromosome 5BL also increases the dormancy, whereas the *TaMKK3* (4AL) had a smaller impact on WGI. Our WGI data suggest an additive effect on dormancy of *TaPHS1* and *TaMKK3* or *TaQsd1*, additivity disappearing when the three genes are combined.

The stronger impact of the 4AL and 5BL QTLs when considering the WGI rather than the SGI may result from the protocol used to characterize the grain dormancy phenotype by Tan et al. (2006). The initial phenotype description based on a germination test (Mares et al. 2002) is comparable to our WGI assay.

Tan et al. (2006) demonstrate a strong genotype × environment interaction for the QTL 4AL but a remarkably consistent effect across environments for the QTL 5BL. This environmental influence, represented in our work by 2 years of field conditions, could explain the higher standard deviations observed in the WGI data for the 4AL lines compared to the 5BL one.

Preexisting constitutional copies of the *TAMKK3* and *TaQsd1* genes in the cultivar Cosinus could explain the small to non-significant effects of these genes in triticale. However, the pyramidization of the same genes in a PHS susceptible wheat breeding line shows very similar results (unpublished data). It is unlikely that both of our recurrent parents contains these two genes. The marker linked to TaMKK3 was validated in several wheat lines derived from Aus1408 (Zhang et al. 2008). However, the *TaQsd1*-related marker was never used to introduce the PHS resistance QTL and could be non-specific.

### Breeding triticale for PHS resistance

Breeding for triticale began in the mid-twentieth century and the first commercial cultivars were released in the 1970s (Oettler 2005). As a relatively small number of wheat and rye accessions resulted in the production of primary triticale, the genetic diversity of current varieties is low (Niedziela et al. 2016). The possibilities of adapting this cereal to market requirements are therefore limited. Initial rusticity of triticale decreases resulting from the expansion of cultivated area inducing the emergence of new races of pathogen continuously adapting to that species (Mascher et al. 2005; Müller et al. 2021). Breeders are now facing new challenges such as improving pest and disease resistance (Mergoum et al. 2009).

Triticale is still considered as a minor cereal crop. The construction of a genetic map is recent (Tyrka et al. 2015) and a small number of QTLs or genes have been identify (Wajdzik et al. 2019, Mergoum et al. 2019; Ollier et al. 2020). Actually molecular breeding remains difficult in triticale.

The production of new wheat-rye hybrid (primary triticale) increases the genetic variability in triticale by introducing new selected genes recently discovered in wheat or rye parental lines. This approach failed to generate performant triticale cultivars because primary triticale shows generally low fertility and poor agronomic performances (Oettler 2005).

To improve genetic diversity, new breeding methods are based on the development of chromosome aberrations though cross-hybridizations (Kwiatek and Nawracała 2018). Translocations and modifications of triticale chromosomes are generated when triticale is crossed with bread wheat (Lukaszewski and Gustafson 1983; Jeberson et al. 2021) or with rye (Lapinski and Rafalski 2003). Rye is a valuable source of genes in wheat breeding by wheat-rye chromosomes translocations (Saulescu et al. 2011; Crespo-Herrera et al. 2017; Moskal et al. 2021) and could also be useful for triticale improvement. Several PHS resistance QTLs in rye were available when this study started (Twardowska et al. 2005, Masojć et al. 2007, Masojć and Milczarski 2009, Myskow et al. 2010). They were not considered as potential candidate for improving PHS resistance in triticale because our objective was to use the same genes and markers in our wheat and triticale breeding programs and wheat genes were preferred.

As wheat is genetically well documented, the creation of wheat × triticale hybrids, with the A and B genomes of wheat as genes pools donors, is a promising strategy to facilitate the introduction of new agronomic traits in triticale breeding program. Recently, this method enables to introduce into the triticale genome the QTLs of resistance to fusarium head blight *Fhb1* and *Qfhs.ifa-5A* from the wheat variety Sumai-3 (Ollier et al. 2020) and the slow-rusting genes *Lr34/Yr18* and *Lr46/Yr19* from wheat germplasm Frontana (Skowrońska et al. 2020). For the first time, this approach results in new performant triticale varieties and two of them (911.55046 and 911.55051) will be registered soon.

In wheat, the effectiveness of *TaPHS1* and *TaMKK3* depends on the variety of wheat they are derived from and on environmental conditions (Lin et al. 2018). This study also recommends the pyramidization of the two genes to increase PHS resistance. Our new cultivars contain three genes for resistance to PHS (*TaPHS1*, *TaMKK3*, and *TaQsd1*) and only the *TaPHS1* is useful. During the early stages of selection, the efficacy of these genes was unknown in triticale and addictive effects was expected. The *TaPHS1* alone is able to generate PHS-resistant triticale cultivars. Unlike disease resistance genes that the pathogen can overcome, *TaPHS1* should provide lasting effects. Resistance to PHS is an important trait in triticale but many other traits could be optimized to create performant cultivars. The number of genes introduces with MAS into a variety exponentially increases the number of samples to be analyzed in order to maintain sufficient genetic diversity. The impact of genes or QTLs on agronomic performances should be significant to compensate the economical MAS effort involved.

## Data Availability

All data are given in the manuscript.

## Code availability

Publicly available software are used in this study.
